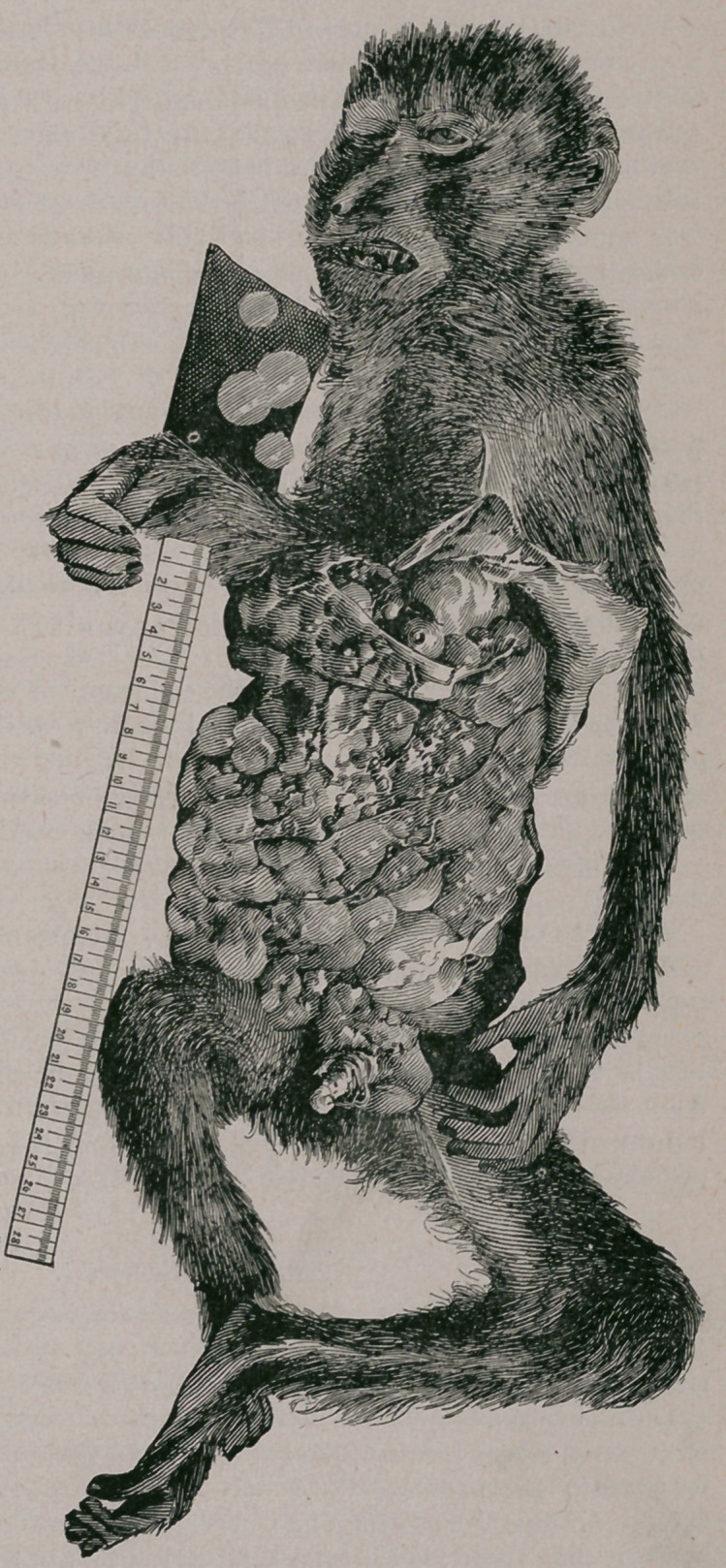# Autopsies at Central Park Menagerie

**Published:** 1886-04

**Authors:** 


### CASE DEPARTMENT.

### AUTOPSIES AT CENTRAL PARK MENAGERIE.

### PIG-TAILED MONKEY (*Macacus Nemestrinus*).

Made Oct. 17th, 1885. Body somewhat emaciated. Rigor mortis moderate.

Abdomen distended and tense, but affording no distinct fluctuation. Upon incision, it was found to contain a large number of cysts, covered by a layer of the omentum, but some escaping from beneath it through some holes which either pre-existed or were produced when the confining tension of the abdominal walls was removed. No ascites. The cysts appear to be nearly all situated within the omentum, and but for ruptures in the latter, might be lifted out of the abdomen within it. There were a few, however, beneath the peritoneum in the pelvis, which they nearly filled. The cysts varied from the size of a hen's egg to that of a pea, and those collected weighed 46 ounces.

The intestines, stomach and kidneys were apparently normal. There were adhesions binding the liver to the diaphragm, right kidney and transverse colon. The right and middle of the three chief lobes of the liver appeared normal but for slight thickening of the peritoneal covering, due to perihepatitis. The left lobe was nearly entirely replaced by a cystic tumor the size of a hen's egg, with tough, opaque walls, which upon section proved to be multilocular.

The lower end of the spleen was involved in a mass of small cysts situated in the gastro-splenic omentum.

When the thorax was opened, the left side presented two large cysts—one above and one below—which nearly filled the thoracic cavity on that side. Between these cysts was the heart, lying in a transverse position, the apex towards the left and rotated, so that the right side of the heart was directed forwards. The lower cyst was behind the left bronchus and the vena cava inferior, and its great bulk to the left of those structures. The bronchus was much elongated (five or six times as long as that on the right side, which appeared to be normal) and compressed, but was still pervious, as shown by injecting water into the trachea with very moderate force (a one-foot head of water was the force employed). The phrenic and pneumogastric nerves showed no signs of compression or injury. The lung on the left side was almost completely compressed below the cyst, between it and the diaphragm, being capable of crepitation over only a small area. It could be partially inflated after being freed from the adhesions which bound it to the diaphragm and the cyst. Within it was discovered an echinococcus cyst about the size of a large pea. The lower of the two large cysts had upon its upper and right aspect a dark reddish layer, wholly unconnected, as far as could be discerned with the unassisted eye, from the lung tissue already described. This, upon microscopic examination, proved to be pulmonary tissue. The inference drawn from the appearances was that the lower of the two large cysts originated within the lung, and by its growth produced compression and atrophy of the lung tissues. The upper cyst was only connected with the lower one by adhesions, and the tissues about it were so altered and adherent that no indication of its original seat could be obtained.

The right lung was here and there atelectatic, but otherwise apparently normal.

Heart, to outward appearances, normal. It was not opened.

The cysts possessed laminated gelatinoid walls, enclosing fluid contents of a clear, transparent, somewhat yellowish character. They were unilocular except the large one in the liver, and possibly one or two others which were broken when found. Several, which were more minutely examined, contained from ten to thirty scolices, each of which possessed thirty to forty hooklets, but no evidence of suckers. They were thought to be echinococcus veterinorum, as described in Birch-Hirshfeld's "Pathologische Anatomie" (1877).

EDWARD K. DUNHAM, M.D.

---

TIGER *(Felis tigris)*.

Full-grown female, December 10, 1885: autopsy ten hours after death.

Rigor mortis slight, adipose tissues wasted in some places—notably the abdominal region—showing gelatinous transformation.

Thoracic organs.—The lungs presented an advanced phase of necrosis, large cavities had formed, filled with putrid gas or air, the latter communicated with the bronchi, the others were, one of them, incopulated on all sides. Another, consisting of a mere bag of pleura, and a little ragged and necrotic lung tissue, lay in such a situation that in the half-dark room it was taken for a hydropic pericardium till an incision, the resulting escape of gas, and the ulcerated cavity left behind, dispelled the illusion. The bronchi were filled with a frothy, foul material, glairy in the larger, and more detritus like in some of the smaller bronchi. The bronchial glands were enlarged, strongly pigmented, and two of them showed a cheesy center. Several of the bronchi of the second sub-division showed bronchiectasis, one such dilatation had a ragged ulcer at its floor. All contained a more or less purulent. glairy material, and the lining membrane, where not ulcerated, was thickened. Scattered throughout the lung substance, which in the middle and upper lobes of both sides was consolidated, there were numerous nodules of a cheesy character, many of which showed signs of breaking up. They were all of them lobular in character, and the bronchus leading to them was inflamed, thickened and usually blocked up. There were a few pigmented pleural adhesions of recent formation.

The right side of the heart was slightly hypertrophied, the valves and endocardium were normal.

Abdominal Cavity.—Gastro-intestinal tract: the stomach and intestines were empty, excepting the colon descendens, which contained normal fœcal masses. There was no evidence of disease. The liver and spleen, which latter was strongly pigmented, were also structurally healthy. The kidneys were intensely congested in both the cortical and medullary portion. The bladder was empty and healthy, as were also the generative organs. There was a large corpus luteum (corpora lutea of pregnancy) in each ovary, distinctly organized, and nearly two centimeters in diameter.

Head.—The brain, which showed no distinct anomaly, was unusually soft in its anterior portion, the blood in all the cerebral vessels was of a serous character.

The membrane lining the right tympani bulbs was greatly thickened, while on the left side it was thin, colorless and transparent. It was here yellowish brown, beautifully injected by a rich net-work of blood vessels, and opaque. The membrane tympanic of this side was also thick and opaque, the membrane lining the ossicula injected.

Post-mortem Diagnosis.—Evidence of a general catarrhal attack of long antecedence—probably a year—localizing especially in the respiratory tract and the right tympani cavity, and resulting in bronchiectasis, atelectasis and catarrhal pneumonia, with terminal formation of vomicæ. ⁂

## Figures and Tables

**Figure f1:**